# Dynamic Interplay of Smooth Muscle α-Actin Gene-Regulatory Proteins Reflects the Biological Complexity of Myofibroblast Differentiation

**DOI:** 10.3390/biology2020555

**Published:** 2013-03-28

**Authors:** Arthur Roger Strauch, Seethalakshmi Hariharan

**Affiliations:** Department of Physiology & Cell Biology and the Ohio State Biochemistry Program, the Dorothy M. Davis Heart & Lung Research Institute, The Ohio State University College of Medicine, Columbus, OH 43210, USA; E-Mail: hariharan.13@buckeyemail.osu.edu

**Keywords:** Myofibroblast, gene transcription, smooth muscle actin, wound healing, fibrosis

## Abstract

Myofibroblasts (MFBs) are smooth muscle-like cells that provide contractile force required for tissue repair during wound healing. The leading agonist for MFB differentiation is transforming growth factor β1 (TGFβ1) that induces transcription of genes encoding smooth muscle α-actin (SMαA) and interstitial collagen that are markers for MFB differentiation. TGFβ1 augments activation of Smad transcription factors, pro-survival Akt kinase, and p38 MAP kinase as well as Wingless/int (Wnt) developmental signaling. These actions conspire to activate β-catenin needed for expression of cyclin D, laminin, fibronectin, and metalloproteinases that aid in repairing epithelial cells and their associated basement membranes. Importantly, β-catenin also provides a feed-forward stimulus that amplifies local TGFβ1 autocrine/paracrine signaling causing transition of mesenchymal stromal cells, pericytes, and epithelial cells into contractile MFBs. Complex, mutually interactive mechanisms have evolved that permit several mammalian cell types to activate the SMαA promoter and undergo MFB differentiation. These molecular controls will be reviewed with an emphasis on the dynamic interplay between serum response factor, TGFβ1-activated Smads, Wnt-activated β-catenin, p38/calcium-activated NFAT protein, and the RNA-binding proteins, Purα, Purβ, and YB-1, in governing transcriptional and translational control of the SMαA gene in injury-activated MFBs.

## 1. Overview and Scientific Scope of the Review

Myofibroblasts (MFBs) are smooth muscle-like cells that provide the contractile force required for tissue repair and remodeling during wound healing. Although transient in the context of normal tissue repair, dysfunctional MFBs that escape clearance and accumulate in healing tissue is a leading cause of chronic fibrotic disease in the cardiopulmonary, renal, and hepatic systems. MFBs arise in proximity to injured epithelial and vascular endothelial beds and have a temporal relationship to the cellular developmental process referred to as epithelial-mesenchymal transition (EMT) required for repair of denuded or damaged basement membranes. Results of lineage-fate mapping studies suggest that MFBs arise *in vivo* chiefly from resident mesenchymal stromal cells and microvascular pericytes in response to paracrine factors secreted by stressed epithelial cells during EMT. Among these factors, the leading agonist for MFB differentiation is transforming growth factor β1 (TGFβ1), deposited by injured epithelial and endothelial cells as well as immune cells that infiltrate sites of tissue damage and inflammation. Via rate-limiting, receptor-regulated Smad 2/3/4 nuclear proteins, TGFβ1 activates transcription of genes encoding smooth muscle α-actin (SMαA) and subunits of type I interstitial collagen that are prototypical phenotypic markers for MFB differentiation. Importantly, non-canonical, Smad-*independent* TGFβ1 signaling additionally activates pro-survival Akt kinase and p38 MAP kinase. Inhibition of GSK3β kinase by activated Akt prevents β-catenin degradation that may be especially important for activating β-catenin-dependent genes such as cyclin D, laminin, fibronectin, and metalloproteinases needed for epithelial cell proliferation, migration, and restoring adhesion of repaired cells to the basement membrane. Importantly, β-catenin occupies a pivotal position in a feed-forward regulatory loop that can enhance expression of TGFβ1 agonist and augment TGFβ1 receptor regulated canonical and non-canonical signaling in injured epithelial cells and mesenchymal stromal cells. While EMT-associated β-catenin signaling can explain transient acquisition of mesenchymal cell-like behavior by damaged epithelial cells, there must be concurrent mechanisms for the specific activation of SMαA and collagen promoters in nearby mesenchymal stromal cells and pericytes, and possibly epithelial cells, to allow their transition into contractile, force-transducing MFBs. This aspect of molecular control will be reviewed with an emphasis on the dynamic interplay between multiple nuclear and cytosolic proteins that collaborate in governing expression of the SMαA gene in injury-activated MFBs.

## 2. Principles and Prototypical Features of Myofibroblast Activation

***TGFβ1 is an essential mediator of MFB differentiation***. Wound healing is a complex series of innate-immune responses that have evolved to limit blood loss while sanitizing and physically repairing sites of tissue injury. A provisional matrix constructed of fibrin protein is established by thrombosis very soon after traumatic injury that stanches blood loss and provides a protein-matrix substrate for infiltration of immune cells needed to combat microbial contamination and initiate tissue reconstructive processes [1,2,3,4]. Immune cells and nearby epithelial and vascular endothelial cells secrete proteases that activate latent TGFβ1 secreted by injured cells in the immediate area as well as deposited in the wound during platelet de-granulation [5,6]. Conformation changes in the TGFβ1 latency complex induced by a combination of physical interaction with a fibronectin-rich basement membrane and specific integrin receptor proteins provide additional TGFβ1-activation capacity in wounded tissue [7,8]. Of particular importance for understanding and controlling cellular dysfunctional behavior in healing wounds is the self-sustaining nature of TGFβ1 activation during MFB differentiation regardless of whether tissue damage is traumatic or the result of more insidious metabolic-stress injury. Newly polymerized actin stress fibers [1] physically engage and alter the conformation of cortical integrin αvβ6 in the epithelium [8] and integrin αvβ5 [9,10] in parenchymal cells that enhances activation of latent TGFβ1. Within minutes after injury, phosphorylation and nuclear translocation of TGFβ1 receptor-regulated Smad proteins [11] bind cognate *cis*-regulatory elements in promoters required for transcriptional activation of genes encoding SMαA [12,13,14] and type I collagen subunits α1 and α2 [15,16]. MFBs secrete interstitial collagens and fibronectin, which replace the soft provisional fibrin network with a more structurally robust and rigid array of matrix protein polymers [9,17,18] referred to as granulation tissue required for sustaining the differentiated MFB phenotype. The developmentally scheduled decrease in granulation tissue cellularity driven in part by apoptosis of activated MFBs marks the end of a normal episode of wound-healing activity [3].

***Consequences of MFB dysfunction***. While SMαA-positive MFBs evolved as transient and beneficial participants in the wound-healing process, chronic MFB differentiation can damage healthy parenchymal tissue in what has been referred to as the medical syndrome of “endless healing” [3]. There are numerous examples of tissue pathology that arise when MFBs do not undergo apoptosis and continue to secrete excessive amounts of collagen resulting in the formation of hypertrophic scar tissue. Fibrosis and endless healing are serious, irreversible complications of diseases associated with cardiac contractile dysfunction and arrhythmia including myocardial infarction and hypertrophic cardiomyopathy [19,20,21,22,23]. Idiopathic pulmonary fibrosis, non-specific interstitial pneumonitis, sarcoidosis, bronchopulmonary dysplasia, and alveolar fibrosis during ALI/ARDS are all characterized by severe, MFB-associated airway remodeling [24,25]. Excessive MFB differentiation and fibrosis also are widely recognized, post-surgical complications of solid-organ transplantation [26,27,28,29,30,31,32]. Cardiac allograft dysfunction, in particular, is a tissue-remodeling abnormality in accepted heart grafts associated with accumulation of SMαA-positive stromal and adventitial MFBs that contribute to interstitial and perivascular fibrosis, accelerated coronary arteriosclerosis, biomechanical-stress injury responses in the myocardium, and premature graft failure [33,34,35,36,37,38]. Unfortunately, efforts to stem inflammatory responses previously believed to be the root cause of MFB differentiation and fibrotic disease have not been effective [24,39]. SMαA is one of the earliest genes to be activated during MFB differentiation and precedes peak expression of type I interstitial collagen by about 1–2 days [40,41]. Therefore, analysis of temporal events associated with activation of SMαA gene expression could help reveal molecular-regulatory checkpoints for possible therapeutic control of the earliest stages of MFB differentiation prior to onset of irreversible fibrotic disease. On-going studies of SMαA gene expression during MFB differentiation in mouse and human have revealed the existence of a complex, auto-regulatory loop under control of the TGFβ1 and thrombin wound-healing agonists with aspects of both transcriptional and translational control [13].

## 3. SMαA Gene Transcriptional Regulation as a Hallmark of Myofibroblast Differentiation

***The origins of MFBs***. Published reports suggest multiple cellular origins for the MFB lineage including resident stromal fibroblasts or progenitor-like cells, circulating fibrocytes migrating to sites of tissue injury following their birth in the bone marrow, and mesenchymal cells arising nearby or directly from injured epithelial or endothelial cell monolayers through the embryologic process referred to as epithelial- (or endothelial-) mesenchymal transition (EMT). The cellular, molecular, and developmental details of EMT pathobiology are complex and will not be reviewed in this article. The reader is directed to excellent overviews of EMT in the recent biomedical-research literature that are especially informative with respect to the cellular biology of wound healing in the pulmonary and renal systems [8,24,42,43]. Regardless of the cellular origins of mature MFBs, TGFβ1 and thrombin are well-accepted fibrogenic agonists often present at the moment of tissue injury and seem to play a direct role in governing expression of mammalian genes encoding the SMαA and type I collagen proteins required for MFB function in a variety of tissue beds and cell types.

**Figure 1:**
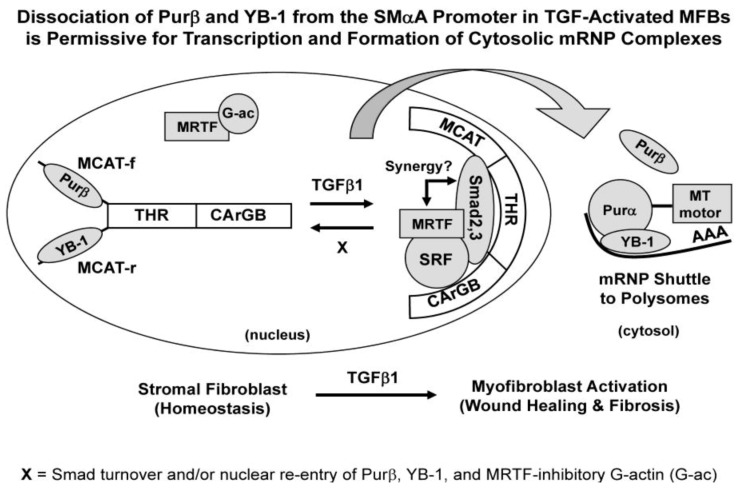
The SMαA promoter in quiescent fibroblasts is occupied by the Purβ and YB-1 repressors that occupy opposite strands of the MCAT/THR transcription-activation site. In the presence of TGFβ1, conformational changes occur in the MCAT/THR accompanied by binding of Smad2/3, SRF, and MRTF at cognate sites within and around this site (*i.e.*, Smads at the THR and MRTF/SRF at CArG box B). MRTF normally is sequestered by abundant G-actin monomer that becomes depleted as the SMαA cytoskeleton forms in TGFβ1-activated myofibroblasts (MFBs). The release of MRTF enables its interaction with SRF to enhance SMαA gene transcription. As repressor proteins are removed from the promoter, they bind exon 3 in the nascent SMαA mRNA transcripts to form cytosolic mRNP complexes for transport to polysomes. Association of the mRNA:Purβ:YB1 complex with Pur α in the cytosol may enable coupling with microtubule motor proteins that mediate mRNP intracellular transport known to be under control of Purα in neuronal cells [60]. In theory, reversal of TGFβ1-mediated MFB activation is possible (denoted by the X) upon turnover of Smads and nuclear re-entry of Purβ, YB1, and G-actin monomer after completion of actin cytoskeleton assembly. For simplicity, only Purβ is shown in the nucleus. However, Purα can bind the same DNA site (MCAT-f). Also, the TEF1 and Sp1 *trans*-activators are omitted from the diagram but are known to bind at the MCAT and downstream SPUR site (not shown), respectively, within the 200 bp SMαA core promoter.

***SMαA gene activators and repressors***. With specific regard in this review article to operation of the mammalian SMαA gene, multiple transcriptional regulatory proteins along with their cognate binding sites within promoter DNA have been identified that respond to biomechanical and metabolic signals generated as a consequence of tissue injury. The mechanism for regulation of the SMαA promoter is based on combinatorial interaction of two primary activating systems under control of constitutively expressed proteins including transcriptional enhancing factor 1 (TEF1), serum response factor (SRF), and specificity proteins 1 and 3 (Sp1/3) plus one or more rate-limiting proteins including the TGFβ1 receptor-regulated Smad proteins 2, 3 and 4, myocardin-related transcription factor A (MRTF-A), and calcium/calcineurin-regulated nuclear-factor-of-activated T-cell protein (NFAT). Operating in opposition to these *trans*-activator proteins is a set of SMαA gene repressor proteins first described by the Getz, Strauch, and Kelm investigative teams [12,14,33,34,44,45,46,47,48,49,50,51,52,53,54,55]. Three DNA-binding proteins with a preference for single-stranded nucleic acid, the purine-rich binding proteins Purα and Purβ plus the stress response-associated Y-box binding protein 1 (YB-1), cooperate and physically interact within the 200 bp SMαA core promoter in a manner that overlaps and potentially interferes with binding sites for the TEF1, SRF, Sp1/3, and Smad *trans*-activators (Figure 1). These transcriptional activators all bind double-stranded SMαA promoter DNA whereas Purα, Purβ, and YB-1 show a clear preference for single-stranded promoter regions and act as repressors [50,54,55]. Although details are beyond the scope of this review, it is interesting to note that several of these DNA-binding proteins, including the Smads, SRF, and YB-1, also are known participants in controlling transcription of genes encoding the α1 and α2 subunits of the type I collagen protein [15,16,56,57].

**Figure 2:**
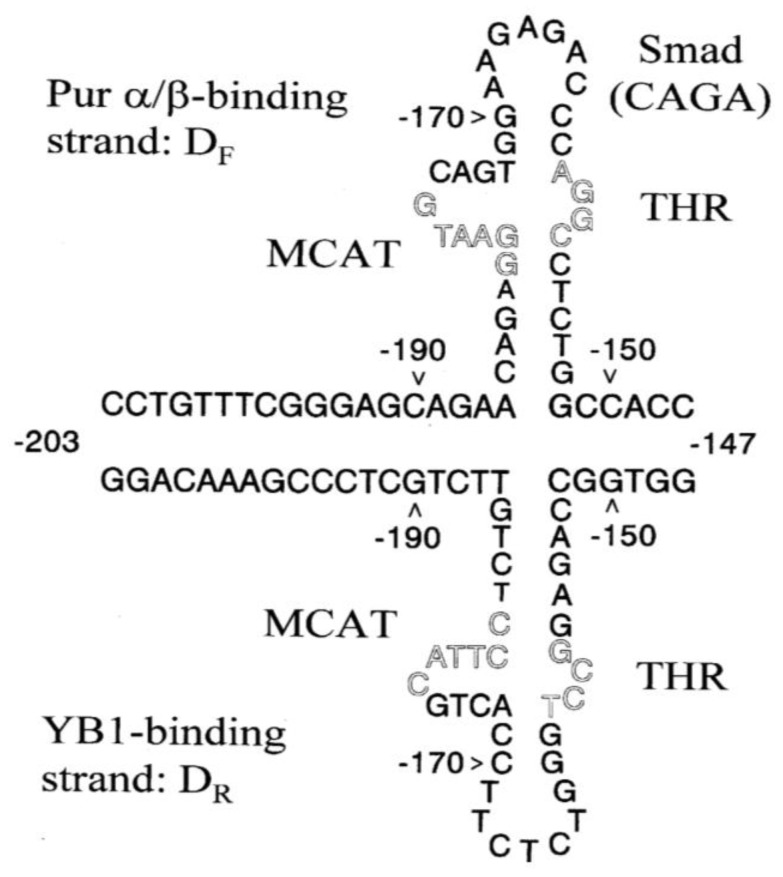
A thermodynamically favorable (ΔG of about −12 kcal/mmol) stem-loop structure can be formed within the MCAT/THR site in the SMαA core promoter. This model depicts formation of single-stranded loops with purine- (D_F_) and pyrimidine-rich (D_R_) asymmetry that bind Pur proteins and YB-1, respectively. Our working hypothesis is that Pur and YB-1 repressor proteins minimize SMαA gene transcription in quiescent stromal fibroblasts by disrupting duplex-DNA sites within the MCAT/THR region encompassing the (a) Smad-binding consensus sequence CAGA and/or (b) TEF1-binding site consensus AGGAATG. TGFβ1 activation of the SMαA promoter during MFB differentiation facilitates removal of the Pur and YB-1 repressors transiently exposing their former purine- and pyrimidine-rich binding sites to chemical modification by reagents specific for single-stranded DNA [49]. Nuclear uptake of TGFβ1-regulated Smads helps eliminate Pur and YB-1 repressor binding to the single-stranded loops [13,14] allowing the cruciform to re-fold into duplex B-DNA necessary for binding and transcriptional activation by Smads and TEF1 within the MCAT/THR. Additionally, MRTF/SRF and Sp1 also may bind at nearby re-folded DNA sites in CArG B and SPUR, respectively (not shown). The positions of MCAT/THR mutations that affect SMαA promoter activity are located in sequences depicted by hollow letters.

***The SMαA core promoter contains a stem-loop control element***. The Pur proteins favor interaction with purine-rich sequence strands whereas YB-1 (also known as MSY-1 in rodent species) has high affinity for pyrimidine sequences in the SMαA promoter. We have shown that competitive interplay between the TEF1 activator and Purα/Purβ/YB-1 repressors can regulate transcription from a region of the SMαA promoter called MCAT/THR that contains an inverted repeat with a high potential to form single-stranded loops within a thermodynamically favorable (ΔG ~ −12 kcal/mmol) cruciform structure (Figure 2). A central feature of SMαA promoter dynamic control is the reversible formation of a stem-loop structure that encompasses the MCAT/THR region within the 200 bp core promoter [49]. Exposure of single-stranded regions within chromatin encompassing the SMαA core promoter was demonstrated experimentally during TGFβ1 stimulation by selective reactivity with chemical reagents that preferentially target unpaired or unstacked DNA structures (chloroacetaldehyde, potassium permanganate) or act as protein-footprinting agents (dimethyl sulfate). These chemical probes previously were shown to be capable of reacting with unusual non-B DNA structures that revealed regions of chromatin protection resulting from protein binding. The MCAT/THR motif was particularly enriched for reactive sites and exhibited chromatin conformational changes in response to TGFβ1 [49]. Of interest, the motif contains multiple Smad2/3 binding sites, represented by the consensus sequence CAGA, as well as purine- and pyrimidine-rich sequences comprising the forward-strand and reverse-strand binding sites for the Pur proteins and YB-1, respectively [58,59].

Although not identified on the basis of reactivity with single-strand specific chemical modifiers, another region of duplex DNA in the SMαA promoter also is affected by TGFβ1 in a manner that alters Pur protein repressor interaction with the Sp1 *trans*-activator proteins. This site called SPUR, so named by its ability to bind both Sp1 and Pur proteins, is located between −59 and −28 in the 5'-flanking region of the SMαA promoter [14]. Notably, a GC-rich sequence called TCE (for TGFβ1 control element) located in the center of SPUR represents a core Sp1-binding site absolutely required for MFB SMαA gene expression *in vitro* and *in vivo* [14,48,61,62]. Sp1 and Pur proteins not only bind to different regions in SPUR but also form a physical complex in the absence of DNA pointing to the possibly additional importance of off-DNA complexes in mediating transcriptional output during MFB differentiation [14]. One proposed mechanism is that Pur repressors physically sequester the Sp1 activator away from the SMαA promoter in the absence of TGFβ1 in the extracellular microenvironment. Notably, protein:protein interactions between Sp1, Pur α, and Pur β were all significantly reduced in TGFβ1-actived MFBs in a Smad 2/3/4-dependent manner [14]. In this regard, the SPUR contains a single CAGA Smad-binding motif at its 3' end. This feature potentially provides a means for phosphorylated Smads to assist Sp1 in *de novo* activation of the SMαA promoter in quiescent stromal fibroblasts possibly by neutralizing and/or displacing pre-bound Pur protein repressors in this segment of DNA. In this regulatory model, Smads occupy and collaboratively activate the SMαA promoter at two sites: the upstream MCAT/THR region mentioned earlier and the downstream SPUR site. Two other SMαA gene repressors, Egr-1 and KLF4, may assist Pur proteins with preventing activator interaction with the promoter by competing with Sp1 for the TCE site within SPUR DNA [12,63].

As *in vivo* proof-of-principle, dynamic interplay of SMαA gene transcriptional activators and repressors was operative during the development of TGFβ1-associated chronic graft fibrosis after murine heterotopic heart transplant [34,64]. Sequestration of SMαA *trans*-activators by Pur protein repressors was reduced in protein extracts prepared from explanted heart grafts in parallel with development of left ventricular fibrosis, which is a hallmark of chronic rejection pathobiology. However, in the heart graft model, SRF rather than Sp1 was the *trans*-activator targeted by Pur protein repressors [34]. Along with activation of SMαA-positive MFBs, the fetal-type SMαA gene also is reactivated in adult cardiomyocytes as a consequence of biomechanical stress generated in the graft myocardium due to deposition of inelastic scar tissue by cardiac MFBs [16,65,66,67]. Reactivation of the SMαA gene after cardiac transplant was accompanied by decreased interaction between the SPUR motif and Pur proteins with an associated increase in binding of SRF to the essential CArG B site in the SMαA promoter [34]. The ability of Pur proteins to occupy and coordinate transcriptional activity at two distinct sites in the 200 bp core promoter provides evidence for the modular nature of SMαA transcriptional control that most likely is fined tuned by combinatorial interaction of activators, repressors, and their cognate DNA-binding sites in a tissue-, disease- and/or MFB developmental stage-specific manner.

***Epigenetic control of SMαA gene expression***. Not much is known about the direct influence of small non-coding RNA molecules on SMαA transcription factor functionality in MFBs nor is it clear if epigenetic modulators that alter DNA methylation, histone acetylation, and protein phosphorylation oversee dynamic changes in chromatin structure encompassing the SMαA promoter locus. Some details are emerging, however, showing that expression of certain microRNA species (miRs) during liver fibrosis can alter Smad4 levels that could impact SMαA promoter activity in MFBs [68]. Additionally, there are reports showing the presence of CpG islands in the promoter and first intron of the SMαA gene that may be involved in DNA methylation-dependent transcriptional silencing [69]. In particular, intronic CpGs are highly methylated in epithelial cells that normally do not express SMαA protein compared to SMαA-permissive fibroblasts. While the precise effect of DNA methylation on interaction of specific transcriptional regulatory proteins with SMαA gene is not known, there is evidence that methyl CpG-binding protein 2 (MeCP2) binds and induces the SMαA promoter when ectopically expressed in fibroblasts [70]. Although a deficiency in this protein in fibroblasts from MeCP2 null mice led to reduced SMαA expression, its absence did not seem to dampen TGFβ1-inducibility of the SMαA gene [70]. These findings are of interest and point to some indirect, modulatory effect of MeCP2 on TGFβ1-induced MFB gene expression independent from its ability to interact with SMαA promoter DNA.

## 4. Interplay between TGFβ1 Signaling and Actin Cytoskeleton Dynamics Governs SMαA Gene Transcriptional Output in Myofibroblasts

***The G-actin pool provides feedback control of SMαA gene transcription***. SMαA gene transcription driven by TGFβ1-regulated Smads can be enhanced during MFB differentiation by collateral signaling generated by increased G-actin monomer polymerization [16,66,71,72]. In pulmonary and renal MFBs, SMαA gene activation based on collaboration between SRF and the myocardin-related transcription co-activator protein MRTF-A (also known as MAL/MKL1) is mediated by actin-cytoskeleton dynamics in the absence of overt TGFβ1-dependent Smad signaling. MRTF-A binds SRF via peptide determinants located in the *N*-terminal region consisting of a basic amino acid region, a short α-helical region, and a Glu-rich domain. A key feature of actin-mediated SMαA promoter activation is the shuttling of MRTF-A into the nucleus in parallel with its dissociation from G-actin monomers that become depleted by the burst of F-actin polymerization during MFB activation. MRTF-A contains at least one and as many as three Arg-Pro-X-X-X-Glu-Leu (RPEL) motifs near the *N*-terminus that mediate physical interaction with G-actin [73,74,75]. Prywes and co-workers discovered an important MRTF-A phosphorylation site and found that an inactivating mutation resulted in constitutive localization to the nucleus suggesting that phosphorylation inhibits nuclear localization [76]. The sequence context of this serine 454 residue resembled an extracellular signal-regulated kinase 1/2 (ERK1/2) phosphorylation site. G-actin binding to MRTF-A promoted nuclear export and MRTF-A phosphorylation was required for binding to G-actin thus explaining the sub-cellular localization behavior of MRTF-A. Although nuclear localization of MRTF-A initially is tied to rapid depletion of the G-actin pool during fast assembly of the F-actin cytoskeleton, transport becomes impaired by Erk1/2-kinase phosphorylation that allows formation and export of MRTF-A:G-actin complexes from the nucleus once actin polymerization subsides and the G-actin pool becomes restored to its normal steady-state size. In a related study on suppression of TGFβ1-inducible SMαA gene activation in MFBs by pro-inflammatory TNFα, increased MEK1/Erk1/2 signaling was identified as a key factor in silencing SMαA gene transcription [12]. Both the Egr-1 and YB-1 transcriptional repressor proteins required Erk1/2 kinase activity for binding and inhibiting the SMαA core promoter. Taken together, these data suggest that pro-inflammatory agonists such as TNFα, IFNγ, and IL-1β previously implicated in inhibiting MFB differentiation and fibrosis [15,77], may augment Erk1/2 signaling that is not only required for removing MRTF-A from the MFB nucleus but also enhancing interaction of the Egr-1 and YB-1 repressors with their cognate transcriptional silencing sites in SPUR and MCAT/THR, respectively.

***Smads fine tune SRF-mediated transcription***. G-actin physically sequesters MRTF-A and precludes formation of an essential nuclear *trans*-activation complex with SRF. Accordingly, activation of SMαA gene transcription can proceed, in theory, via biomechanical signaling alone simply mediated by changes in the rate of G-actin polymerization and expansion of the actin cytoskeleton during MFB differentiation. This mechanism is less dependent on availability of paracrine factors in the wounded area such as TGFβ1, although this growth factor certainly can initiate MFB differentiation and amplify the overall response. Elberg and co-workers reported that TGFβ1 did not have a discernable effect on MRTF-A expression or nuclear compartmentalization [72]. MRTF-A over-expression regardless of TGFβ1 availability was sufficient to induce SMαA expression in renal tubular epithelial cells although TGFβ1 seemed to facilitate binding of SRF/MRTF-A protein complexes to CArG box elements in the SMαA promoter. Disassembly of cell-cell contacts in renal epithelial monolayers by calcium depletion also was observed to enhance nuclear retention of MRTF-A via Rho/Rho kinase- and Rac-dependent modulation of actin cytoskeleton structure [78,79,80]. Although downstream Smad3-binding sites located between positions −57 to +28 in the SMαA promoter appeared to be dispensable for expression in TGFβ1-activated epithelial cells, more upstream Smad3-binding sites located in the MCAT/THR region that undergoes robust chromatin conformational change in the presence of TGFβ1 have not yet been examined for Smad3/MRTF/SRF binding in these cells. On the other hand, Qiu *et al.* reported that physical interaction between Smad3 and SRF is, in fact, required for TGFβ1-dependent activation of the gene encoding SM22α, also known as transgelin, a 22 kDa protein that shares sequence homology with calponin and bundles F-actin to facilitate the formation of stress fibers needed for MFB contractility [81,82].Kapus and co-workers point out that changes in Smad3/MRTF/SRF dynamic interplay may be cell-context specific and function as a clock to define temporal stages of epithelial cell-response to injury. For example, the mesenchymal cell-to-MFB transition may begin with the initial repression of E-cadherin gene transcription (the Smad3-dominant phase) and conclude with expression of end-stage MFB markers (the MRTF-dominant phase) such as SMαA, SM22α, and interstitial type I collagen [78,79]. Quite likely there will be disease- and/or tissue-specific variations in transcription factor interplay and mechanisms operative in pulmonary lower airway MFBs may differ from those that govern SMαA gene activation in the renal interstitium. In this regard, cardiac and pulmonary fibroblasts appear to utilize SRF in collaboration with Smads to activate the SMαA promoter whereas embryonic stromal fibroblasts rely on interplay between Smads, TEF1, and Sp1/3 *trans*-activators [14,34,48].

**Figure 3:**
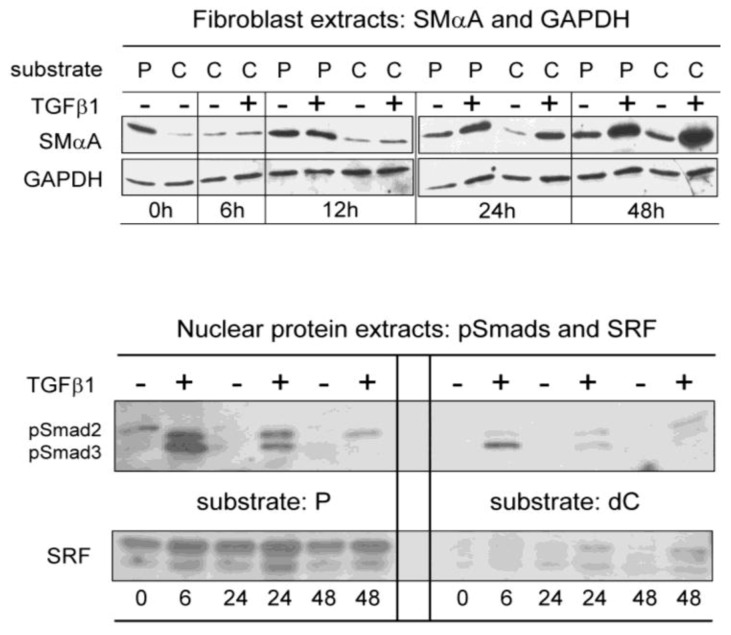
Substrate-dependent control of SMαA expression in human pulmonary fibroblasts. Cultivation of pulmonary fibroblasts on rigid, plastic (P) substrates in low-serum culture medium sustained baseline expression of SMαA over a 48 h period (***upper panel***). Treatment with TGFβ1 (5 ng/mL) amplified SMαA expression due to robust signaling provided by accumulation of nuclear stores of phosphorylated Smad 2/3 (***lower panel***). In contrast, cells maintained on native (C) or denatured type I collagen (dC) substrates showed less baseline SMαA expression during the initial 24 h period and exhibited relatively muted accumulation of phosphorylated Smads 2 and 3 after treatment with TGFβ1 (***lower panel***). The potentiating effect of rigid-plastic substrate conditions on baseline expression of SMαA correlated with high nuclear levels of SRF *trans*-activator protein in the apparent absence of TGFβ1-regulated Smad signaling (***lower panel***). SMαA expression on rigid plastic previously was associated with actin stress fiber assembly in pulmonary fibroblasts [13,92].

***Mechanotransduction influences SRF-mediated transcription***. MRTF/SRF signaling is a useful checkpoint device during wound healing that puts SMαA gene transcription under direct control of actin filament assembly required for MFB contractility. In this regard, we and others have determined that up-regulation of SMαA protein expression in stromal fibroblasts can occur in the complete absence of TGFβ1 signaling simply in response to substrate conditions that are favorable for assembly of actin stress fibers. An interesting report by Sandbo *et al.* outlines a triphasic model for MFB differentiation that involves aspects of both transcriptional and post-transcriptional control [83]. An initial Smad-dependent transcriptional event early after cellular injury when active TGFβ1 is abundant culminates in expression of Rho GTPase. Subsequently, there is a delayed, post-transcriptional response whereby newly synthesized Rho and its associated Rho kinase mediate G-actin polymerization with resultant nuclear accumulation of MRTF-A/SRF SMαA gene-activating complexes. Although the last aspect of the model is not yet fully understood, there seems to be a final, feed-forward step involving *de novo* transcription of the MRTF-A gene possibly driven by SRF itself and not dependent on Smad signaling *per se* [83]. In this regard, we have observed that cultivation of human pulmonary fibroblasts on a rigid-plastic substrate sustained high-baseline expression of SMαA that was clearly evident in the absence of TGFβ1 yet readily amplified if this agonist was provided to the cells over a 48 h treatment period (Figure 3). In contrast, cultivation of fibroblasts on collagenous substrates (either native or denatured forms of type I collagen) markedly delayed the spontaneous expression of SMαA for up to 48 h and TGFβ1 was required for full expression of SMαA. Of interest, fibroblasts maintained on plastic were highly enriched in nuclear stores of SRF compared to cells maintained on either denatured collagen (Figure 3) or native collagen (data not shown). As a possible explanation for robust nuclear accumulation of SRF in fibroblasts maintained on rigid plastic, others have shown in studies of cardiac fibrosis that biomechanical stretch mediated by rigid scar tissue coupled with β1 integrin signaling via an integrin-linked kinase (ILK) was sufficient to induce SRF and MRTF-A expression [16,66,84,85,86,87]. Indeed, Smad-independent, delayed nuclear MRTF/SRF signaling as proposed by Sandbo *et al.* [83] may be fully sustained by permanent scar tissue long after TGFβ1 levels have subsided in the wounded region. Prolonged elevation of nuclear MRTF/SRF would sustain excessive transcription of SMαA mRNA and production of G-actin that while possibly beneficial for early stage healing could escalate into MFB dysfunction if not properly controlled. ILK may play an instrumental role in guiding sub-cellular localization and polymerization of newly synthesized G-actin as it reportedly helps establish actin filament connections at integrin attachment points along the cell membrane. In view of its function as a scaffold protein and cellular-stretch sensor, ILK may coordinate rapid translation of SMαA mRNA with subsequent polymerization of G-actin monomers at the fast end of F-actin anchored to MFB focal adhesions. One of the ILK-associated adaptor proteins, PINCH, has been shown to form a complex with the G-actin-binding protein, thymosin β4 which is required for repairing myocardial injuries [85]. The TGFβ1 type I receptor kinase as well as the pro-fibrotic agonist thrombin *via* its cognate PAR1-dependent GPCR signaling [88] can both activate members of the Rho GTPase family of proteins that fine tune G-actin polymerization kinetics and route spatial deployment of F-actin into either diffuse cortical networks or rigid stress fibers [89,90]. Following successful completion of wound contraction and closure, actin filament depolymerization in MFBs increases the size of the G-actin pool providing the means to automatically sequester MRTF-A in the cytosol and bring transcription of the SMαA gene to closure.

Regulation of actin filament dynamics specifically required for MFB contractility seems to have evolved in parallel with signaling mechanisms that communicate biomechanical information from the extracellular milieu directly to the SMαA gene transcriptional machinery via the integrin family of matrix protein receptors [91,92]. Accordingly, clinical control of cell-substrate signaling in MFBs associated with fibrotic diseases such as scleroderma may help offset dysfunctional feed-forward amplification of SMαA transcription due to excessive nuclear uptake of MRTF-A in response to unchecked G-actin polymerization. Recent reports indicate that accumulation of hypercontractile MFBs in scleroderma may result from the abnormally high rate of actin filament polymerization in these cells due to over-activated focal adhesion kinase [93]. Importantly, therapeutic interventions aimed at controlling focal adhesion kinase (FAK) activity using the FAK/src inhibitor PP2 or the selective FAK inhibitor PF-573,228 were able to attenuate SMαA gene expression in scleroderma fibroblasts [93]. The consequences of these treatments on the level or DNA-binding activity of transcriptional regulatory proteins such as MRTF-A and SRF have not yet been examined but certainly of interest in future investigations.

## 5. Modulation of SMαA Promoter Activity in Mesenchymal Stromal Cells via Crosstalk between Wnt and TGFβ1 Signaling Pathways

***Cell surface injury diversifies Smad protein interactions in MFBs***. While TGFβ1-regulated Smad proteins directly activate the SMαA promoter, their ability to interact with other signaling proteins that accumulate during EMT suggests that their function can be shaped to perform other tasks during MFB differentiation not necessarily related to the biogenesis of a specialized contractile apparatus. During pulmonary EMT, a nuclear-protein complex is formed between TGFβ1-regulated Smad2 and a tyrosine-phosphorylated form (Y654) of the β-catenin transcriptional regulatory protein that is the primary downstream mediator of Wingless/int (Wnt) developmental signaling [8]. Formation of the Smad2:pY654β-catenin heteromeric complex requires: (a) α3β1 integrin to mediate β-catenin phosphorylation in the presence of TGFβ1 and (b) treatment with low-calcium medium formulated to weaken epithelial cell-cell contacts and allow dissociation of β-catenin from E-cadherin cell surface complexes [94,95]. Activated TGFβ1 available at the site of tissue injury has been described as a necessary “second-hit” signal that potentiates low calcium-induced uncoupling of epithelial cell junctions by providing the Smad protein partner for β-catenin [43,79].

In damaged epithelial cells, nuclear β-catenin activates the T-cell factor 1/lymphoid enhancer factor 1 (TCF1/LEF1) regulatory complex required for mobilizing transcription of genes encoding a variety of basement membrane-repair proteins [96]. Convergence of Wnt and TGFβ1 signaling pathways via formation of a protein complex between their respective downstream mediators, β-catenin and Smad2, may be especially important during EMT for the transcriptional regulation of genes needed for the proliferation and survival of stress-injured epithelial cells. However, this collaborative interaction has not been implicated in governing transcription of the SMαA gene. Regardless of its phosphorylation status, Smad2 does not bind SMαA promoter DNA (Hariharan and Strauch, unpublished data) yet EMT has been linked to enhanced SMαA gene transcription in both pulmonary and renal epithelial cell models. This mystery has been resolved to some extent by recent reports showing that dispersed epithelial cells from both lung and kidney sources also generate phosphorylated Smad3:β-catenin nuclear protein complexes. Smad3-containing β-catenin complexes do in fact appear to bind SMαA promoter DNA and also seem to indirectly mediate additional promoter activation by the MRTF/SRF complex [78,97]. Moreover, reports on bronchopulmonary dysplasia (BPD) support the idea that Smad activation of the SMαA gene also may be amplified by β-catenin in mesenchymal stromal cells thus extending the potential importance of this regulatory scheme beyond the epithelial cell lineage [96]. In human neonatal BPD, alveolar septa are thickened with collagen protein and infiltrates of SMαA-positive MFBs [98]. In early passage stromal cells from infants at risk for developing BPD, there was a direct relationship between the levels of inactive phosphorylated GSK3β, active β-catenin, and accumulation of SMαA protein [96]. Transduction of neonatal lung fibroblasts with a constitutively active form of GSK3β blocked their ability to undergo MFB differentiation due to the ability of the active GSK3β to degrade the pool of available cytosolic β-catenin. Accordingly, over-expression of a degradation-resistant, truncated form of β-catenin in fibroblasts was sufficient for MFB differentiation.

***Novel regulation of SMαA promoter activity by crosstalk between Smad3 and β-catenin***. Among the genes that can be activated by β-catenin signaling are those related to basement membrane repair, cell migration, and angiogenesis such as fibronectin, laminin-γ2, matrix metalloproteinases, versican, and VEGF. As mentioned earlier, corresponding stiffening of the local extracellular matrix due to ongoing deposition of collagen and fibronectin can influence actin cytoskeleton dynamics and directly drive MRTF/SRF-mediated activation of the SMαA promoter potentially obscuring any separate contributions provided by nuclear β-catenin or β-catenin:Smad complexes. Authentic binding sites for the β-catenin-dependent TCF1 and LEF1 transcription factors presumably are absent in the SMαA and COL1α2 promoters and neither gene is listed in the Stanford University DNA-sequence compilation of canonical Wnt/β-catenin signaling targets [99]. Nevertheless, β-catenin could amplify the binding and/or *trans*-activation potency of Smad2, Smad3, and/or MRTF within the SMαA promoter by forming physical complexes with these or other transcriptional regulatory proteins subsequent to TGFβ1 activation. In this regard, two recent reports have highlighted the potential importance of EMT-associated accumulation of nuclear β-catenin in injured pulmonary [97] and renal epithelial cells [78] especially with regard to how its interaction with Smad3 influences SMαA promoter activity. Studies from the Kapus group revealed a critical role for Smad3 in altering the stability of MRTF in porcine (LLC-PK1) and murine (rat NRK-52E) proximal renal tubule cells [78]. Disruption of renal tubule epithelial cell monolayers by reduction of physiologic calcium in the presence of TGFβ1 fostered β-catenin interaction with Smad3 with two important consequences. Sequestration of Smad3 away from MRTF enhanced formation of the MRTF/SRF transcriptional activation complex at an essential CArG box element in the SMαA promoter. In addition, β-catenin enhanced MRTF protein stability thereby preserving MRTF/SRF SMαA gene *trans*-activator availability by preventing the Smad3-mediated recruitment of GSK3β that regulates MRTF ubiquitination and degradation. In this capacity, β-catenin operates as a de-repressor in renal epithelial cells through its ability to form an off-DNA complex with Smad3 that would otherwise limit the extent of SRF-mediated SMαA gene activation by eliminating MRTF. Whether similar strategies are operative in mesenchymal stromal cells, pericytes, or epithelial cells in the context of MFB differentiation and fibrosis will be important subjects for further research inquiry.

A different approach reported by Borok and co-workers showed that physical interaction between SMαA promoter DNA and a ternary complex of β-catenin, Smad3, and CBP (a cyclic AMP-responsive element-binding protein (CREB)-binding protein) was sufficient to mediate SMαA expression in TGFβ1-activated rat RLE-6TN pulmonary epithelial cells [97]. Interestingly, chromatin immunoprecipitation assays revealed that the Smad3:β-catenin protein complex exhibited site-specific binding and was detected only on the upstream Smad-binding element (SBE1) located in the SMαA promoter between −552 and −513 but not another site (SBE2) located in the 5'-flanking region between −5 and +28. While the upstream SBE1 is located in a 700–800 bp segment of the SMαA promoter previously shown to be essential for smooth muscle- and MFB-specific transcriptional activity [1,58,59,100], the Smad3-binding site located in the MCAT/THR region that exhibits TGFβ1-dependent chromatin conformational changes in MFBs [49] has not yet been examined for its ability to bind Smad3:β-catenin protein complexes. The formation of these novel protein complexes may have clinical relevance because immunoreactive β-catenin and Smad3 were identified in both nuclear and peri-nuclear compartments of hyperplastic type II alveolar epithelial cells adjacent to fibroblast foci in lung sections from patients with idiopathic pulmonary fibrosis [97].

***Epithelial-mesenchymal “interaction” is distinct from epithelial-mesenchymal “transition”***. While epithelial cells in tissue culture microenvironments appear to be capable of expressing SMαA protein in the context of EMT-associated injury, it also is important to note that canonical Wnt signaling via the GSK3β/β-catenin/TCF1/LEF1 axis has been shown to directly activate TGFβ gene transcription [101]. In native tissue context, this could result in widespread production and secretion of this pro-fibrotic agonist by injured epithelial cells with subsequent paracrine-activation of nearby mesenchymal stromal cells and pericytes causing their conversion into SMαA-positive MFBs. Collateral damage to the basement membrane including altered functionality of the α3, αvβ5, and/or αvβ6 classes of integrins would amplify regional MFB activation by converting latent stores of TGFβ1 released by injured epithelial cells into biologically active ligand. Wnt-mediated production of activated TGFβ1 not only would stimulate transcription of genes encoding prototypical MFB-specific markers such as SMαA and type I collagen subunits, but also stimulate pro-survival Akt and MAP kinases with subsequent phosphorylation and inhibition of the GSK3β gatekeeper enzyme. Inactive GSK3β cannot mediate the proteolytic degradation of β-catenin and the resultant activation of β-catenin-dependent genes such as cyclinD, matrix metalloproteinases, and various basement-repair proteins can mediate the cellular hyperplasia and migration aspects of EMT [102]. But perhaps more importantly, concurrent TGFβ1 signaling and nuclear uptake of Smad3, with or without β-catenin involvement, would trigger SMαA gene transcription enabling nearby mesenchymal stromal cells and perhaps even damaged epithelial cells themselves to cease proliferation and transition into contractile MFBs.

Recent reports highlight the complex relationship between Wnt and TGFβ1 signaling pathways that transpire in both epithelial cells and stromal fibroblasts in the context of chronic fibrotic disease. For example, the Wnt ligands Wnt5a and Wnt3a activate TGFβ1 signaling in both embryonic fibroblasts [103] and intestinal epithelial cells [104]. In particular, Wnt3a enhanced SMαA expression in fibroblasts by up-regulating Smad2 expression via a β-catenin-dependent mechanism [103]. TGFβ1 exerted a 2-step, feed-forward effect on MFB differentiation by augmenting expression of the Wnt11 agonist in renal epithelial cells [105] while decreasing expression of the Wnt signaling antagonist Dickkopf-1 in dermal fibroblasts via non-canonical p38 signaling [106]. Finally, prominent activation of the canonical Wnt pathway with nuclear accumulation of β-catenin in diverse samples of fibroblasts from patients with idiopathic pulmonary fibrosis or liver cirrhosis suggests that activation of Wnt signaling in stromal cells may be a general feature of human fibrotic disease [106].

## 6. Non-Canonical TGFβ1 Receptor Signaling: Calcium-Calcineurin Control of SMαA Gene Regulation during Myofibroblast Differentiation

***Wound healing agonists augment intracellular calcium***. Tissue-specific transcription of the mammalian SMαA promoter in vascular and enteric smooth muscle cells as well as differentiated MFBs depends on the presence of an intact intron 1 sequence containing tandem SRF, nuclear factor of activated T cells (NFAT), and AP1 transcriptional activator protein-binding sites [34,58,107,108,109]. The physiologic importance of these intronic sequences was not known until recently when it was discovered that G-protein coupled receptor (GPRC) signaling under control of several wound healing agonists including thrombin, angiotensin II, and endothelin-1, caused calcium influx via enhanced expression of the transient receptor potential canonical protein family member, TRPC6 [110]. Calcineurin, a calcium-activated protein phosphatase, dephosphorylates and enhances nuclear uptake of the NFAT transcriptional activator protein that has been suspected in the regulation of SMαA gene activity [21,111]. Central to this scheme was an upstream role for p38 MAP kinase activated by both GPCR and TGFβ1RI signaling that enabled expression of the *trpc6* gene product required for calcium influx, calcineurin/NFAT interplay, NFAT nuclear translocation, and transcriptional synergy with SRF within the first intron of the SMαA promoter. Calcineurin-dephosphorylated NFAT has rather weak affinity for its AGGAAA consensus sequence (+1,106 to 1,111) and requires SRF binding to an adjacent CArG box (+1,098 to 1,107) to activate target gene promoters [107].

While fibroblasts lacking the *trpc6* gene have an impaired wound healing response, calcium agonists appear to elicit different responses depending on the tissue source used for MFB isolation [107,111,112]. In this regard, it is unclear if NFAT/SRF interplay in the context of elevated intracellular calcium is a universal feature of SMαA gene activation in MFBs in the manner of the SRF/Smad activation scheme that operates in the 5'-flanking region of the promoter. Moreover, it is not known if the calcium/NFAT/SRF activation signal is disease-stage specific nor is there information regarding the physical occupancy of SMαA intron 1 sequences by NFAT/SRF in native chromatin context. Paradoxically, when over-expressed in constitutively active form, NFAT attenuated endothelin-1-induced expression of SMαA protein in cardiac MFBs [112]. As an explanation, cardiac MFB differentiation elicited by endothelin-1 might be a two-stage process with aspects of both feed-forward and feed-back regulation to better control accumulation and transient participation of MFBs during the wound healing process. Endothelin-1 initially stimulated MFB differentiation via Gα_12/13_ signaling but subsequent up-regulation of the TRPC6 gene by the same G protein species increased basal calcium influx activity yet blocked MFB accumulation [112].

***Calcium-dependent and -independent modes of SMαA transcriptional control***. Signaling related to regulation of calcium homeostasis may have complex effects on genes required for MFB differentiation owing to its essential role outside the nuclear compartment in controlling actin-based contractility and integrin-mediated cell adhesion. As reviewed earlier, actin filament dynamics and cell adhesion directly influence SMαA gene transcription via the MRTF/SRF promoter-activating complex. Calcium control of NFAT/SRF interplay at the intronic AGGAAA/CArG site may operate in parallel with the calcium-insensitive MCAT/THR and CArG box B elements located in the SMαA 5'-flanking region [34,107]. However, the intronic control module may provide a unique calcium-responsive feature necessary to initiate MFB differentiation in the context of a healing-wound microenvironment that is highly enriched for GPCR agonists such as thrombin [88], angiotensin II [112,113], endothelin-1 [112] as well as reactive oxygen intermediates such as NADPH oxidase-induced peroxide [114] that all facilitate release of intracellular calcium stores. Re-establishment of intracellular calcium homeostasis following an episode of wound healing may provide a potent inhibitory signal to down-regulate MFB differentiation thereby preventing unchecked progression to permanent scar formation. In this regard, the concentration of endothelin-1 needed to induce Rac1/ROS/SRF-mediated SMαA expression in cardiac MFBs was about 10-fold lower than that needed to elevate TRPC6 expression, calcineurin/NFAT signaling, and calcium-dependent SMαA gene repression [112]. This observation suggests that MFBs may have acquired an auto-regulatory ability to limit calcium-dependent responses and thus minimize progression to fibrosis in the presence of multiple wound-healing agonists such as thrombin, angiotensin II, endothelin-1, and TGFβ1. Alternatively, high-calcium, calcineurin/NFAT-dependent signaling may up-regulate expression of genes encoding anti-fibrosis agents such as bone morphogenic protein 2 (BMP2), a potent TGFβ1 antagonist [115], or enhance secretion of inflammatory cytokines such as IFNγ and/or TNFα that increase nuclear levels of YB-1 and Egr-1 repressors proteins that block activation of the SMαA promoter [12].

***Non-canonical TGFβ1 signaling in MFBs is provided by p38 MAP kinase***. Not withstanding the controversy regarding the precise role of calcium and NFAT in mediating SMαA gene transcription during MFB differentiation, evidence presented by Molkentin and co-workers provides compelling evidence that p38-mediated calcineurin expression seems to be necessary for proper healing of dermal wounds in mice [111]. A variety of experiments using TGFβ1-activated cardiac and dermal fibroblasts under conditions where experimental deficiencies in TGFβ1 receptor signaling or TRPC6 expression were implemented revealed a lesser importance for canonical Smad signaling and emphasized the essential nature of p38 MAP kinase in deployment of the SMαA cytoskeleton. Interestingly, SRF knock-down in dermal fibroblasts prevented SMαA-filament polymerization in the presence of TGFβ1 or angiotensin II, which could be rescued by over-expression of the TRPC6 calcium channel protein. These results suggest that SRF provides an essential upstream signal necessary for TRPC6 gene transcription and calcium-regulated MFB differentiation, at least as measured by assembly of a functional SMαA cytoskeleton. Evidence that TRPC6 can directly activate the SMαA promoter by enhancing the DNA-binding activity of the calcium-responsive NFAT/SRF activator complex has not yet been presented in the literature. When constitutively-active calcineurin was over-expressed in virally-transduced cardiac fibroblasts, only 50% of the cells exhibited SMαA stress fibers suggesting that not all the cells were able to respond in this manner to an excess of NFAT-activating phosphatase [111]. The relatively prolonged activity of p38 MAP kinase generated by non-canonical TGFβ1 signaling is in marked contrast to nuclear uptake of phosphorylated Smads that occurs within 1–2 h after TGFβ1 receptor activation. Coupled with the observation that polymerization of SMαA stress fibers, and not transcription *per se*, was inhibited by deletion of the primary p38 target (MAP kinase-activated protein kinase 2; [111]), it is interesting to speculate that p38 MAP kinase specifically regulates deployment of SMαA filaments at some post-transcriptional and/or post-translational level of control in calcium-activated MFBs. Indeed, pro-fibrotic thrombin, angiotensin II and endothelin-1 that do not recruit Smad transcriptional activators may preferentially enhance p38 MAP kinase signaling and associated intracellular calcium leak through their cognate GPCR pathways. Calcium accumulation therefore appears to be an important rate-limiting event during MFB differentiation with primary importance in assembly of the SMαA cytoskeleton needed for contractility. Accordingly, therapeutic targeting of the TRPC family of ion channel proteins may offset calcium dysfunction and prevent excessive accumulation of contractile MFBs as a maladaptive response to the presence of multiple fibrogenic agonists at the site of tissue injury.

As a final note, in a manner similar to biomechanical-stress injury caused by deposition of rigid scar tissue, hyperosmolarity in kidney tubule epithelium alters actin cytoskeleton dynamics that results in cell shrinkage and shape deformation. Interestingly, p38 MAP kinase in hyperosmotic stress-injured epithelial cells functions as a transcription antagonist and seems to enhance proteosomal degradation of nuclear MRTF needed for SRF-mediated activation of the SMαA promoter [116]. Paradoxically, p38 MAP kinase can enhance SRF phosphorylation and stabilize its ability to activate certain gene promoters especially those associated with the early response to mitogen stimulation and expression of genes needed for cell cycle control. Taken together, the available data suggests that p38 kinase may have dual functions in wound healing depending on whether newly activated MFBs are dividing and require phosphorylated SRF for transcription of cell cycle control genes, or more mature and assembling the SMαA contractile apparatus that may specifically require the calcium-handling aspects of p38 signaling.

## 7. The Emerging Model for Post-Transcriptional Control of SMαA Gene Expression: Thrombin-Mediated Regulation of mRNA-Binding Proteins

***Thrombin regulates SMαA mRNA translation in MFBs***. Along with the regulation of G-actin polymerization, there is another equally important post-transcriptional mechanism for governing SMαA protein expression in MFBs that operates at the level of mRNA translational control. Pro-fibrotic mechanisms that drive tissue repair after traumatic injury have developed under strong evolutionary pressure to rapidly stanch blood loss, close open wounds, and restore capillary beds needed for oxygen delivery. Accordingly, accumulation of SMαA mRNA in the cytosol may increase wound repair efficiency by providing priority access to polysomes for rapid translation and localized deployment of the actin cytoskeleton required for MFB contractility. Further supporting the notion of “fast-track” control over the MFB differentiation process, TGFβ1 is abundantly stockpiled in the extracellular space in a latent state awaiting rapid activation at the moment of tissue injury by the combined action of proteolysis and integrin-mediated mechanotransduction. During periods of ischemic stress associated with wound healing and tissue remodeling subsequent to myocardial infarction, IPF, cirrhosis, and renal allograft vasculopathy, there also appears to a mechanism that allows for preferential translation of mRNAs encoding proteins required for cellular adaptation to low-oxygen conditions [117,118].

Thrombin is a ubiquitous serine protease that not only initiates the enzyme cascade responsible for blood coagulation but also activates stores of latent TGFβ1 and mediates MFB differentiation and tissue remodeling in both native and transplanted heart, lungs, and kidneys [119,120,121,122]. Our studies of syngeneic murine heart grafts have revealed that serial transplant surgery with repeated bouts of ischemia/reperfusion injury fosters myofibroblast accumulation and severe fibrosis via a molecular process that involves collaboration between TGFβ1 and thrombin [33,34]. Examination of the regulatory basis for increased SMαA expression in MFBs revealed that while TGFβ1 induced *de novo* transcription of the SMαA gene, thrombin augmented SMαA mRNA translational efficiency [13]. Thrombin displaced both YB-1 and Pur proteins from exon 3 coding sequences in SMαA mRNA previously shown to mediate translational silencing. Within five minutes after exposure to thrombin, cytosolic YB-1 was consolidated within the nucleus and a striking increase was observed in deployment of cytoplasmic SMαA thin filament networks. The type I TGFβ1 receptor serine/threonine kinase inhibitor SB431542 substantially reduced SMαA protein accumulation in TGFβ1-treated fibroblasts but had no negative effect on induction by thrombin [13]. Likewise, a thrombin serine protease inhibitor prevented SMαA protein accumulation but did not block induction by TGFβ1. Therefore, the ability of thrombin to rapidly augment SMαA protein synthesis does not seem to involve amplification of an underlying TGFβ1-dependent process such as activation of latent TGFβ1 or increased mRNA transcription. The MAP kinases Erk1,2 are known to mediate aspects of PAR-1 thrombin-receptor signaling in vascular smooth muscle cells [123]. In this regard, the MEK1 inhibitor U0126 prevented Erk phosphorylation and nuclear uptake of YB-1, increased the size of the cytosolic YB-1 pool, and blocked accumulation of SMαA protein in thrombin-treated pulmonary fibroblasts [13]. The data suggested that rapid elevation of SMαA protein synthesis in hPFBs following exposure to thrombin was based on an Erk-mediated mechanism that fostered highly efficient translation of pre-existing SMαA mRNA. 

***Coordination of SMαA transcription and translation in MFBs by an mRNA shuttle***. The release of YB-1 from SMαA promoter DNA coupled with its ability to bind SMαA mRNA and shuttle between the nucleus and cytosol is suggestive of a regulatory loop for coordinating SMαA gene output in MFBs at both the transcriptional and translational levels. YB-1 is known to influence mRNA function in several ways including unfolding secondary structural elements in mRNA as well as recruiting other proteins required for ribonucleoprotein (RNP) packaging, transport, turnover, and/or translation [124,125,126,127]. Although less well studied, Pur proteins also have been identified as important linker proteins for tethering mRNA to microtubules and motor proteins as well as transport of 1000S mRNA:protein granules in other cell types [60,128,129,130,131]. As depicted in Figure 1, the release of YB-1 and Pur proteins from chromatin in TGFβ1-actived MFBs and subsequent translocation of these protein to the cytosol and then back to the nucleus as mediated by thrombin-mediated MEK1/Erk1,2 signaling provides compelling evidence for their duel roles in coordinating SMαA gene expression in MFBs at both the transcriptional and translational levels [13]. Thrombin activates a variety of growth-, inflammation-, and wound healing-associated genes and promoter regions flanking these genes often contain binding sites for YB-1 [132]. Moreover, data from Mertens and co-workers also showed that YB-1 directly activates transcription of Smad7, a physiologic Smad2/3 antagonist and potent inhibitor of type I collagen gene transcription [56]. On the other hand, while YB-1 nuclear re-uptake ultimately might terminate transcription of the TGFβ1-dependent SMαA and COL1α1/α2 genes [15,57], other genes that control cell proliferation and adhesion such as PDGF-B [133] and MMP-2 [134] are activated by YB-1. Nuclear flux of the YB-1 protein may provide an effective means to coordinate migration of dividing MFB progenitor cells with deployment of the SMαA cytoskeleton and collagen matrix in mature, stationary MFBs to better position the generation of contractile force within the wound provisional matrix.

## 8. The Pur Protein/YB-1 Complex: A Novel Tool for Governing mRNA Transcription and Transport to Assure MFB Transience during Wound Healing?

***Regulatory-protein interplay in the nucleus and cytosol***. Purβ is a single-strand specific DNA-unwinding protein previously shown to bind and inhibit the SMαA promoter during arterial smooth muscle phenotypic modulation [44,45,46]. Purβ also forms physical complexes with both SRF and Smad proteins as well as with its larger companion protein, Purα, that exhibits weaker binding affinity to the SMαA core promoter. Of interest is the possible role of Purα in facilitating mRNA transport in MFBs based on its well-documented ability to shuttle mRNA molecules from the nucleus to peripheral dendritic tips in neuronal cells [60,128]. Purβ also binds YB-1 [46,50] and the protein complex formed between these two proteins is required to target them to their individual forward-(Purβ) and reverse-(YB-1) strand binding sites in the MCAT/THR region of the SMαA promoter during periods of transcriptional repression (Figure 2). In quiescent fibroblasts or mesenchymal stromal cells, Purβ and YB-1 repressors would be expected to bind SMαA promoter DNA and disrupt the duplex binding sites required for occupancy by TGFβ1-regulated, phosphorylated Smad3. Downstream in the SMαA promoter at the SPUR activation motif, Pur proteins interact with an essential GGA motif and perhaps interfere with SRF binding to the CArG B box element located slightly upstream of SPUR on an adjacent loop of chromatin DNA [14]. Off-DNA complexes between SMαA activators and repressors also have been identified in quiescent fibroblasts [14]. Purα seems to be a highly abundant sequestering protein for SRF in these cells and, unlike nuclear Purβ, seemingly confined to the cytosol based on immunohistochemical analysis of fibrosis following heart transplant [34]. We speculate that as fibroblasts become activated by TGFβ1, phosphorylated Smads enter the nucleus and initiate the process of displacing Purβ and YB-1 from their cognate promoter-binding sites possibly by competitive displacement via formation of protein:protein complexes that weaken binding of gene repressors to the SMαA promoter DNA. Concurrently, the appearance of nascent SMαA mRNA in the nucleus may provide a high-affinity, exon 3-binding site for Purβ and YB-1 that sequesters these proteins away from chromatin DNA [51]. Once removed to the cytosol coupled to mRNA payload, Purβ encounters Purα as a preferred protein-binding partner. The relative stability of the Purβ:Purα complex is higher than the Purα:SRF complex so the potent Purβ repressor probably becomes functionally sequestered in the cytosol allowing the nucleus to accumulate additional stores of free SRF needed for transcriptional activation. The purpose of Purα may be to indirectly potentiate activation of SMαA gene transcription in MFBs by sequestering Purβ repressor in the cytosol thereby freeing SRF for nuclear uptake. Since its affinity for Purβ is greater than that for SRF, the role of Purα may switch from SRF antagonist to SRF co-activator. TGFβ1-regulated Smads coupled with synthesis and polymerization of G-actin monomers into stress fibers and the associated release and nuclear uptake of MRTF-A may augment SRF action and amplify transcriptional output from the SMαA promoter. Subsequent silencing of SMαA transcription and MFB differentiation may be accomplished in the fully healed wound by normal attrition of phosphorylated Smads via protein ubiquitination coupled with nuclear re-entry of Purβ and YB-1 that have been released from the now depleted pool of SMαA mRNA. Reduction in the level of SMαA mRNA following the burst in SMαA protein synthesis and F-actin polymerization represents a natural means to reduce sequestration of Purβ and YB-1 in the cytosol permitting their re-localization to the nucleus to assist in gene repression. The Purβ and YB-1 repressor proteins unfold the MCAT/THR region of the promoter thus de-activating the Smad-binding sites and forcing removal of Purα from the promoter to allow it to sequester any remaining nuclear and cytosolic stores of SRF. We have discovered that Purα has about 10-fold less affinity for the SMαA core promoter DNA compared to Purβ (Hariharan and Strauch, work in progress) suggesting that re-occupancy by Purβ may override any residual binding by Purα thereby freeing up the latter to bind and sequester SRF in the now quiescent cells.

**Figure 4:**
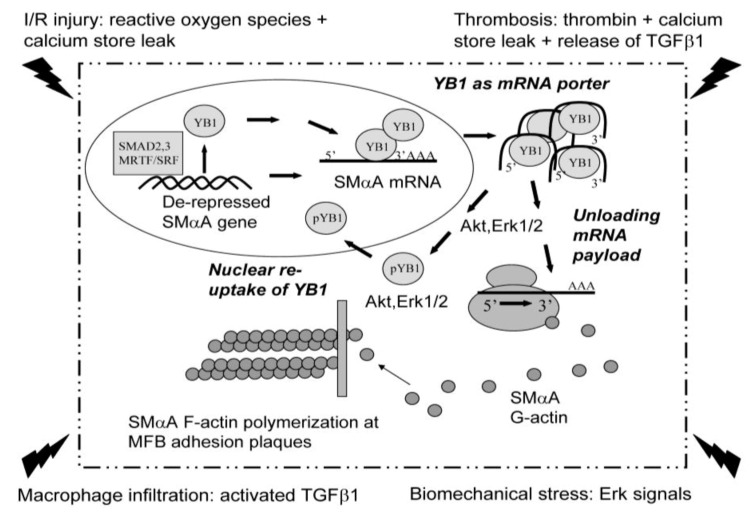
YB-1 may function as a porter for mRNA transport in stress-activated MFBs. Tissue injury-associated events including ischemia/reperfusion, thrombosis, inflammation, and biomechanical stress all can trigger MFB differentiation by increasing the levels of active TGFβ1, intracellular calcium, and MAP kinase signaling. These metabolic signals conspire to de-repress the SMαA promoter by removing YB-1 and Pur proteins from the nucleus (for simplicity, only YB-1 is shown in this scheme). With assistance from Purα, displaced YB-1 can function as an mRNA porter to move nascent transcripts from the nucleus to cytosolic polyribosomes where kinases such as Erk1/2 and Akt may phosphorylate YB-1. Phosphorylation of serine 102 in the RNA-binding cold-shock domain could facilitate both unloading of mRNA payload and nuclear re-entry of YB-1 for additional cycles of SMαA mRNA transport or transcriptional suppression depending on whether MFB differentiation is underway or nearing termination. Newly translated G-actin monomers made available by the YB-1 mRNA shuttle are used to polymerize actin stress fibers at focal adhesions needed for directing MFB contractile force in the healing wound.

***The mRNA porter may utilize microtubules for transport***. Post-transcriptional mechanisms that modify mRNA stability and/or translational efficiency provide rapid and flexible control of gene expression that may be particularly important in coordinating not only the initiation but also prompt resolution of wound-healing responses [135]. The hypothetical scheme for coordinating SMαA mRNA transcriptional and translational responses during MFB differentiation provides aspects of both feed-forward and feed-back control that would be necessary to tightly regulate both the accumulation and timely regression of contractile MFBs. Failure to autoregulate MFB differentiation could explain excessive accumulation of these cells in endless healing syndromes associated with fibrosis and dysfunctional tissue remodeling. Highlighting the multi-functional roles of proteins that coordinate SMαA transcription and translation during MFB differentiation, there now is considerable evidence in the literature showing that Purα and YB-1 can mediate aspects of mRNA packaging and intracellular transport from the nucleus to sites of protein synthesis on cytosolic polyribosomes. YB-1 has been identified as a constituent of so-called “stress granules” where it binds and stabilizes mRNAs that encode proteins needed for cellular adaptation during periods of metabolic stress due to hypoxia, exposure to environmental toxins, or presence of chemotherapeutic agents such as doxorubicin [118,125,127,136,137,138,139,140]. A recently discovered property of YB-1 in the post-transcriptional deployment of viral RNA (vRNA) in mammalian cells infected with the influenza virus adds yet another task for stress granule-associated YB-1 in the process of mRNA transport [141]. Following infection, YB-1 serves as a porter that directs viral ribonucleoprotein (RNP) complexes to microtubules for sub-cellular transport to vesicles where viral-exporting complexes are packaged and combined with vRNA and structural proteins for eventual shedding from infected cells. Taken together, published data suggest that YB-1 may function as a general molecular gatekeeper in MFBs where it controls mRNA pool size and selectively dispatches stored transcripts to polyribosomes for fast-track biosynthesis of specialized proteins such as SMαA needed for prompt repair of injured tissue beds. Unloading of SMαA mRNA payload from YB-1 in proximity to polyribosomes might be required for high-output production of G-actin monomer needed to form contractile thin filament networks in stress-activated MFBs (Figure 4). Recent discoveries in the neuroscience field indicate that YB-1 may be assisted by Purα, an important motor protein-associated factor in neuronal cells whose dysfunctional behavior has been linked to abnormal development and degeneration of the central nervous system [128]. Purα also has been identified as a constituent of transported messenger RNP complexes in *Drosophila* oocytes analogous to its putative role in human neuronal cells [142]. Purα tethers 1000S RNPs to the cytoskeleton to permit long-distance axonal transport from nucleus to dendrites where it accumulates with the Map2 microtubule associated protein and Staufen, a dendritic-branching protein specifically associated with the polyribosome-enriched branch points in neuronal cells [60,130,143]. Notably, microtubule disruption using nocodazole removed Purα from dendrites and re-localized this protein on axons. Of note, Purα is co-localized with YB-1 at sites of sarcomere thin filament remodeling proximal to polyribosome-enriched intercalated discs in ischemia/reperfusion-injured ventricular cardiomyocytes in accepted heart grafts [33,34]. YB-1:Purα heteromeric protein complexes also have been observed in TGFβ1-activated human pulmonary MFBs (Willis, Hariharan, and Strauch, unpublished data). An additional key observation was that Purα forms a high-salt resistant complex with the kinesin-motor protein, KIF5, and can be observed to move bi-directionally in dendrites possibly indicating a tug-of-war for mRNA payload between kinesin and opposing microtubule motors such as dynein [144]. Taken together, the available data suggest that Purα may represent an adaptor molecule for coupling YB-1-bound mRNA to motor proteins to allow transport along the MFB cytoskeleton from packaging sites in the nucleus to cytosolic polyribosomes for expedited protein synthesis.

## 9. Future Directions for Therapeutic Control of MFB Differentiation

**Figure 5:**
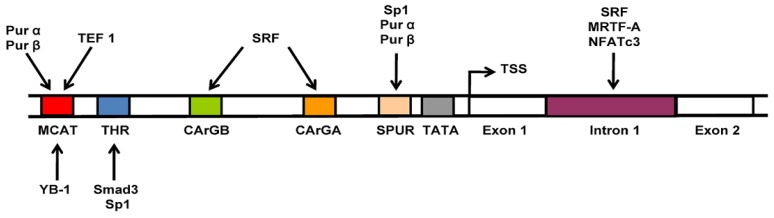
A diagram summarizing key transcriptional regulatory protein-binding sites in the 3.6 kilobase mammalian SMαA promoter including relative positions of consensus sites in the 5'-flanking and first intron regions. Purα, Purβ, and YB-1 are repressors whereas all other indicated proteins are activators. The exact positions of intron 1-binding sites for SRF and NFATc3 have not yet been identified. TSS refers to transcription-start site. Not drawn to scale.

Microarray approaches have been used to catalog and classify constituents of the wound-healing transcriptome [29,145] but virtually nothing is known about dynamic interplay between specific gene activators and repressors that govern mesenchymal cell gene expression and set the pace for MFB differentiation. There is an unmet medical need for therapeutic interventions aimed at controlling rate-limiting steps in MFB differentiation in the clinical background of organ transplant, obstructive pulmonary disease, myocardial infarction, heart failure, liver cirrhosis, interstitial renal disease, and hypertension [1,8,24,43]. Diseases associated with faulty SMαA gene expression and excessive accumulation of MFBs and rigid scar tissue all may exhibit dysfunctional TGFβ1 and thrombin signaling that alters the expression and functional deployment of a variety of DNA- and mRNA-binding proteins that engage in complex interplay and coordinate aspects of transcription, translation, and actin-cytoskeleton dynamics. Recent studies point to the clinical importance of controlling excessive adhesive signaling in MFBs associated with fibrotic diseases such as scleroderma that appear to be based on faulty integrin-mediated activation of focal adhesion kinase [93]. Corresponding changes in the integrity of actin filaments attached to focal adhesions in hypercontractile scleroderma MFBs could directly amplify SMαA gene transcription by altering nuclear translocation of MRTF/SRF complexes that bind and activate the SMαA promoter. In this regard, pharmaceutical agents capable of attenuating focal adhesion kinase catalytic activity and impairing MFB contractility may provide additional benefits by reducing baseline SMαA gene transcription driven by actin polymerization. New drugs have entered clinical trials based on their potential for blocking various steps in TGFβ1 signaling and fibrosis [24,146] but additional opportunities for therapeutic intervention might derive from a better understanding of YB-1, Purα and Purβ with regard to their specific roles in governing MFB response to pro-fibrotic agents such as TGFβ1, thrombin, angiotensin II, endothelin-1, and Th-2 cytokines such as IL-13 to avoid progression to hypertrophic scarring. YB-1 and its companion Pur proteins exhibit unique RNA-binding properties that are likely to be critical in the post-transcriptional regulation of SMαA and type I collagen mRNAs in nascent MFBs. The granulation tissue microenvironment contains both pro-fibrogenic and anti-fibrogenic/pro-inflammatory agonists and it will be important to establish how the primary TGFβ1 activation signal is terminated during MFB differentiation. In this regard, YB-1 and Pur proteins may provide unique regulatory benefits by coordinating mRNA transcriptional output with the packaging, stabilization, and translation of transcripts needed to sustain a transient state of MFB differentiation that lasts no longer than necessary to assure complete healing. The documented ability of TGFβ1 and thrombin to manage the DNA-, RNA-, and protein-binding properties of YB-1, Purα and Purβ provides a useful counterpoint to balance the action of DNA-binding proteins such as Smads, SRF, and Sp1 that activate SMαA and type I collagen subunit gene transcription (Figure 5). Further investigation of dynamic interplay between gene activators and repressors could provide new opportunities for interventional control of MFB differentiation well before the onset of tissue-destructive scar formation.
